# Clinical and functional characterisation of a recurrent *KCNQ1* variant in the Belgian population

**DOI:** 10.1186/s13023-023-02618-4

**Published:** 2023-01-31

**Authors:** Ewa Sieliwonczyk, Maaike Alaerts, Eline Simons, Dirk Snyders, Aleksandra Nijak, Bert Vandendriessche, Dorien Schepers, Dogan Akdeniz, Emeline Van Craenenbroeck, Katleen Knaepen, Laura Rabaut, Hein Heidbuchel, Lut Van Laer, Johan Saenen, Alain J. Labro, Bart Loeys

**Affiliations:** 1 Center of Medical Genetics, Faculty of Medicine and Health Sciences, Antwerp University Hospital, University of Antwerp, Antwerp, Belgium; 2 Medical Genetics (MEDGEN), GENCOR, Faculty of Medicine and Health Sciences, University of Antwerp, Antwerp, Belgium; 3 Experimental Neurobiology Unit, Department of Biomedical Sciences, University of Antwerp, Antwerp, Belgium; 4 Department of Cardiology, Faculty of Medicine and Health Sciences, Antwerp University Hospital, University of Antwerp, Antwerp, Belgium; 5 Cardiovascular Research, GENCOR, Faculty of Medicine and Health Sciences, University of Antwerp, Antwerp, Belgium; 6 Department of Basic and Applied Medical Sciences, Faculty of Medicine and Health Sciences, Ghent University, Ghent, Belgium

**Keywords:** *KCNQ1*, LQTS, Jervell-Lange-Nielsen, Recurrent mutation, iPSC-CMs

## Abstract

**Background:**

The c.1124_1127delTTCA p.(Ile375Argfs*43) pathogenic variant is the most frequently identified molecular defect in the *KCNQ1* gene in the cardiogenetics clinic of the Antwerp University Hospital. This variant was observed in nine families presenting with either Jervell-Lange-Nielsen syndrome or long QT syndrome (LQTS). Here, we report on the molecular, clinical and functional characterization of the *KCNQ1* c.1124_1127delTTCA variant.

**Results:**

Forty-one heterozygous variant harboring individuals demonstrated a predominantly mild clinical and electrophysiological phenotype, compared to individuals harboring other *KCNQ1* pathogenic variants (5% symptomatic before 40 years of age, compared to 24% and 29% in p.(Tyr111Cys) and p.(Ala341Val) variant carriers, respectively, 33% with QTc ≤ 440 ms compared to 10% in p.(Tyr111Cys) and p.(Ala341Val) variant carriers). The LQTS phenotype was most comparable to that observed for the Swedish p.(Arg518*) founder mutation (7% symptomatic at any age, compared to 17% in p.(Arg518*) variant carriers, 33% with QTc ≤ 440 ms compared to 16% in p.(Arg518*) variant carriers). Surprisingly, short tandem repeat analysis did not reveal a common haplotype for all families. One *KCNQ1* c.1124_1127delTTCA harboring patient was diagnosed with Brugada syndrome (BrS). The hypothesis of a LQTS/BrS overlap syndrome was supported by electrophysiological evidence for both loss-of-function and gain-of-function (acceleration of channel kinetics) in a heterologous expression system. However, BrS phenotypes were not identified in other affected individuals and allelic *KCNQ1* expression testing in patient-specific induced pluripotent stem cell-derived cardiomyocytes (iPSC-CMs) showed nonsense mediated decay of the c.1124_1127delTTCA allele.

**Conclusions:**

The c.1124_1127delTTCA frameshift variant shows a high prevalence in our region, despite not being confirmed as a founder mutation. This variant leads to a mild LQTS phenotype in the heterozygous state. Despite initial evidence for a gain-of-function effect based on in vitro electrophysiological assessment in CHO cells and expression of the *KCNQ1* c.1124_1127delTTCA allele in patient blood cells, additional testing in iPSC-CMs showed lack of expression of the mutant allele. This suggests haploinsufficiency as the pathogenic mechanism. Nonetheless, as inter-individual differences in allele expression in (iPSC-) cardiomyocytes have not been assessed, a modifying effect on the BrS phenotype through potassium current modulation cannot be excluded.

**Supplementary Information:**

The online version contains supplementary material available at 10.1186/s13023-023-02618-4.

## Background

Congenital long QT syndrome (LQTS) is an inherited cardiac arrhythmia, which manifests as a prolongation of the corrected QT interval (QTc) on a 12-lead electrocardiogram (ECG) [1]. Pathogenic variants in the *KCNQ1* gene constitute the most frequent genetic etiology for LQTS and are identified in up to 50% of patients (type 1 LQTS or LQTS1) [2]. *KCNQ1*, together with its main auxiliary subunit *KCNE1*, encode the subunits that form the voltage-gated cardiac potassium ion channel responsible for the generation of the slowly activating delayed rectifier potassium current (*I*_*Ks*_) [3, 4]. *I*_*Ks*_ is a major determinant of the action potential duration, and as such the length of the QTc interval [5]. Complete absence of *I*_*Ks*_ in individuals with homozygous or compound heterozygous pathogenic variants in *KCNQ1* leads to LQTS combined with deafness (Jervell-Lange-Nielsen syndrome), while individuals harboring heterozygous pathogenic variants in *KCNQ1* display LQTS with normal hearing.

In general, type 1 LQTS patients express a milder phenotype with a lower QTc to arrhythmia risk ratio compared to type 2 and type 3 LQTS, arising from pathogenic variants in the *KCNH2* and *SCN5A* genes, respectively [6]. The contribution of asymptomatic carriers with normal QTc intervals to populations with established heterozygous pathogenic *KCNQ1* variants is estimated at approximately 30–60% [1, 7]. The observed phenotypical variability of type 1 LQTS can, at least partly, be attributed to the causative variant. Allele-specific effects have been demonstrated clinically in large populations of individuals harboring the same variant, either due to recurrent mutations [8] or inheritance from a common ancestor (founder mutation) [8–12]. The South-African p.(Ala341Val) founder mutation is a well-known example of the allele-specific effect, with a more severe clinical course observed for this variant compared to individuals harboring other pathogenic variants in *KCNQ1* [8]. Additionally, transmembrane and established dominant-negative variants in *KCNQ1* have been associated with a more severe clinical and electrophysiological phenotype compared to haploinsufficiency [13].

Functional assessment of *KCNQ1* variants in vitro offers an interesting opportunity for risk stratification by unravelling the allele-specific effect. Typically, this relies on an electrophysiological characterization in heterologous expression systems [14]. This is especially performed for missense variants, as nonsense variants are expected to lead to haploinsufficiency due to their degradation via nonsense mediated mRNA decay (NMD) mechanisms. As *KCNQ1* RNA can be detected in blood [9, 15], it is technically possible to assess gene expression by whole blood RNA extraction. However, it is unclear whether tissue-specific effects can play a role in mechanisms such as splicing and NMD escape. More recently, induced pluripotent stem cell derived cardiomyocytes (iPSC-CMs) have emerged as an alternative model, which can be applied to both expression studies and functional characterization [16].

In this paper we describe the identification of a novel pathogenic variant in exon 8 of *KCNQ1*, c.1124_1127delTTCA p.(Ile375Argfs*43), in nine different families. We performed haplotype analysis to examine whether the variant was inherited from a common ancestor. Additionally, we characterized the effect of the variant, both clinically, by gathering medical data on homozygous and heterozygous variant harboring individuals, and functionally, by assessing its expression in blood and patient-derived iPSC-CMs and by performing electrophysiological studies. This paper highlights the importance of in-depth variant characterization, as we show that an incomplete assessment could have led to misleading conclusions on the functional effect of this specific *KCNQ1* variant.

## Methods

### Variant identification and haplotype analysis

Probands harboring the c.1124_1127delTTCA p.(Ile375Argfs*43) variant (ENST00000155840 transcript) were identified by Sanger sequencing of all exons of the *KCNQ1* gene (one proband presenting with Jervell-Lange-Nielsen syndrome), or our in-house next-generation-sequencing gene panel (PED MASTR Plus Assay), which encompasses 51 genes associated with LQTS (including *AKAP9*, *ANK2*, *CACNA1C*, *CALM1*, *CAV3*, *KCNE1-2*, *KCNH2*, *KCNJ2*, *KCNJ5*, *KCNQ1*, *NOS1AP*, *SCN4B*, *SNCN5A* and *SNTA1*) and other inheritable primary arrhythmias (n = 8 LQTS probands, additional genetic variants are summarized in Additional file 1: Table S1) [17]. Family members were identified by cascade screening performed by Sanger sequencing. Overall, c.1124_1127delTTCA p.(Ile375Argfs*43) constituted the most frequently observed (likely) pathogenic variant in all probands (n = 27) identified with *KCNQ1*-related LQTS in the cardiogenetics clinic of the Antwerp University Hospital (9/27 (33%)).

**Fig. 1:**
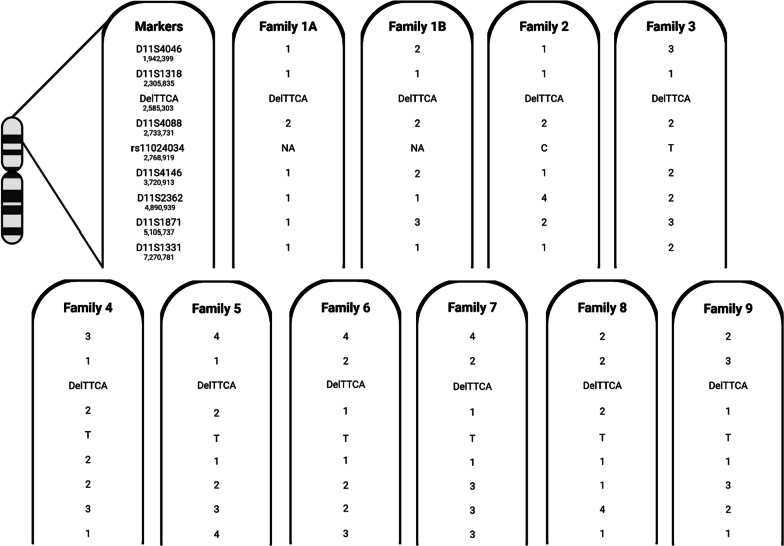
Haplotype analysis of the nine families. NA, not available. Genomic starting locations on chromosome 11 according to Human Dec. 2013 (GRCh38/hg38) Assembly are given below the markers and the variant

Genomic DNA was extracted from blood of at least one family member of the nine families. For the family with two siblings presenting with Jervell-Lange-Nielsen, both parents of the probands were included in the haplotype analysis (Fig. 1, family 1A and 1B). Seven polymorphic short tandem repeats (STRs) flanking the *KCNQ1* c.1124_1127delTTCA p.(Ile375Argfs*43) variant were selected with Map viewer (NCBI): D11S4046, D11S1318, D11S4088, D11S4146, D11S2362, D11S1871 and D11S1331. Primers were designed with Primer3 and labeled with 6-fluorescein amidite (FAM). PCR products were analyzed on an ABI3130XL (Applied Biosystems) in the presence of an internal sizing standard (ROX). Amplicon sizes were determined using ABI GeneMapper software v3.7 (Applied Biosystems). Additionally, one intragenic single nucleotide polymorphism (SNP) within exon 12 of the *KCNQ1* gene (rs11024034) was assessed based on the PED MASTR Plus Assay data. Data on the rs11024034 SNP is lacking for one family (family 1A and 1B, Fig. 1), where the c.1124_1127delTTCA p.(Ile375Argfs*43) variant was detected by Sanger sequencing of all *KCNQ1* exons, as this analysis was outsourced. It was not possible to assess the SNP by other sequencing methods in this family.

### Clinical evaluation

Clinical data of 41 heterozygous and two homozygous (siblings) *KCNQ1* c.1124_1127delTTCA p.(Ile375Argfs*43) variant harboring individuals from nine families were gathered during their follow up at the Antwerp University Hospital. Symptoms were defined as a history of either sudden syncope or sudden cardiac death (SCD). The QTc interval was either extracted from the patient’s medical history, or, when ECG recordings were available, calculated based on the Bazett formula. If multiple ECGs were available, the maximal QTc value was retained. The QTc of one heterozygous carrier was unknown, as this individual died prior to cardiac examinations. 24-h ECG registrations with QTc measurements were gathered for 26 heterozygous carriers. Exercise stress test results were available for 30 heterozygous carriers.

To compare the disease severity induced by the *KCNQ1* c.1124_1127delTTCA p.(Ile375Argfs*43) variant with other *KCNQ1* variants, data was extracted from papers on three *KCNQ1* founder populations [8, 11, 12], and from patients with different (likely) pathogenic *KCNQ1* variants, described in Crotti et al. [8], or genetically diagnosed in our center.

### Electrophysiological studies

Chinese hamster ovary (CHO) cells were cultured in 60 × 15 mm culture dishes (Corning, Tewksbury, MA), with F-12 Ham medium supplemented with 10% fetal bovine serum and 1% penicillin/streptomycin. Cells were grown under a humidified 5% CO_2_ atmosphere at 37 °C. Transient transfections were performed with c.1124_1127delTTCA p.(Ile375Argfs*43) *KCNQ1* cDNA and/or wildtype *KCNQ1* cDNA (1 μg of each construct if co-expressed or 2 μg if expressed alone), supplemented with 1 μg *KCNE1* cDNA and 0.5 μg eGFP using a lipofectamine 3000 transfection kit (Invitrogen). Subconfluent cell cultures were trypsinized 24 h after transfection. Following trypsinization, isolated cells were suspended in F-12 Ham medium and used for electrophysiological recordings within 8 h. For these measurements, only eGFP fluorescent cells were used.

Patch-pipettes were pulled from 1.2-mm borosilicate glass capillaries (World Precision Instrument, Inc., Sarasota, FL) using a p-2000 puller (Sutter Instruments, Novato, CA) and afterwards heat polished. Pipettes with a resistance of 1MΩ–2MΩ were used for the recordings, filled with an intracellular solution of (in mM): 110 KCl, 5 K_2_ATP, 2 MgCl_2_, 10 HEPES and 5 K_4_BAPTA, adjusted to a pH of 7.2 with KOH. The extracellular solution consisted of (in mM): 145 NaCl, 4 KCl, 1 MgCl, 1.8 CaCl_2_, 10 HEPES and 1 glucose, adjusted to a pH of 7.35 with NaOH. Current recordings were controlled with Pclamp10 software using an Axopatch 200B amplifier (Axon Instruments, Foster City, CA) and stored using a Digidata 1440A data acquisition system (Axon Instruments). The data was analyzed with Clampfit software (Axon Instruments). Recordings with a voltage error of > 5 mV after series-resistance compensation were excluded from data analysis.

### Gene expression studies in blood and iPSC-CMs

Whole-blood RNA of two of the heterozygous variant carriers from different families was extracted from a PAXgene® Blood RNA Tube (BD Biosciences) and used for cDNA synthesis with the SuperScript II Reverse Transcriptase kit (ThermoFisher). Exon 8 of *KCNQ1* was amplified by a PCR reaction (with forward primer *TTTGCCATCTCCTTCTTTGC* in exon 7 and reverse primer *ACAGACTTCTTGGGTTTGGGG* in exon 9) and the product was used for Sanger sequencing with the same primers.

Fibroblasts were cultured from skin biopsies of one heterozygous *KCNQ1* c.1124_1127delTTCA p.(Ile375Argfs*43) variant carrier (one of the two individuals who had previously undergone whole-blood RNA extraction) and maintained in RPMI supplemented with 15% FBS, 1% sodium pyruvate, 0,1% Primocin and 1% Penicilline/Streptomycine at 37 °C and 5% CO_2_. Reprogramming to iPSCs was performed with the CytoTune™-iPS 2.0 Sendai Reprogramming Kit and Essential 8 Flex media (Life Technologies), according to the manufacturers’ protocol. Immunocytochemistry staining of the iPSCs showed the presence of pluripotency markers Nanog, Oct4, Tra1-60 and Tra1-81 and RT-PCR proven absence of Sendai virus vectors [18]. Trilineage differentiation potential of the iPSCs was confirmed by embryoid body formation followed by qPCR showing expression of endoderm, mesoderm and ectoderm markers. Genomic integrity was evaluated by copy number variant (CNV) analysis on SNP array data and no significant CNVs were observed.

Differentiation to iPSC-CMs was performed according to a protocol adapted from Burridge et al. [18, 19]. Briefly, mesodermal differentiation was induced by CHIR99021 (6 μM, Axon Medchem) in cardiomyocyte medium consisting of RPMI1640 (Life Technologies) media supplemented with 2% B27 supplement without insulin (Life Technologies) on day 0 for 48 h. On day 2, cardiomyocyte medium supplemented with Wnt-C59 (2 μM, SelleckChem) was added to the cells for 48 h. Next, cells were maintained in cardiomyocyte medium, with addition of T3 starting from day 8. Lactate treatment was performed from day 14 till day 20. Puromycin was added at a concentration of 0.2 mg/ml for 18 h prior to RNA extraction (performed at 34–40 days after initiation of differentiation). RNA was extracted from iPSC-CMs with the Quick-RNA miniprep kit (Zymo Research) and used for cDNA synthesis with the SuperScript II Reverse Transcriptase kit (ThermoFisher). Exon 8 of *KCNQ1* was amplified and sequenced as described for whole-blood derived cDNA.

## Results

### Variant classification and haplotype analysis

The c.1124_1127delTTCA p.(Ile375Argfs*43) variant was initially identified in a homozygous state in two brothers presenting with deafness and *LQTS* (Jervell-Lange-Nielsen syndrome). The variant was classified as pathogenic based on the following American College of Medical Genetics and Genomics and the Association for Molecular Pathology (ACMG/AMP) criteria: null variant (PVS1), extremely low frequency in control databases (1/251.100 according to GnomAD, PM2) [20], co-segregation with disease in multiple affected family members in a gene definitively known to cause the disease (PP1) and patient’s phenotype or family history is highly specific for a disease with a single genetic etiology (i.e. Jervell-Lange-Nielsen syndrome, PP4) [21].

Despite selecting closely nearby STRs at < 0.28 mega base pairs (Mb) upstream and < 0.15 Mb downstream of the c.1124_1127delTTCA p.(Ile375Argfs*43) variant (D11S1318 and D11S4088, respectively), we found that this region was only partially shared for the nine families (Fig. 1). This was also the case for the intragenic rs11024034 SNP located at < 0.19 Mb downstream from the variant. We were therefore unable to confirm the founder mutation status.

### Clinical characteristics

Clinical data was gathered for two homozygous and 41 heterozygous variant harboring individuals (Table 1, Additional file 1: Table S1). Both homozygous siblings were deaf, one of them had a history of syncope. The maximum resting QTc measured in the homozygous siblings was 527 ms and 467 ms (at age 13 and 20 years old, respectively). Both parents, harboring the c.1124_1127delTTCA p.(Ile375Argfs*43) variant in a heterozygous state, also manifested QTc prolongation and were treated with beta-blockade. Out of the eight heterozygous LQTS probands, one male individual (13%) was diagnosed after dying suddenly at rest at the age of 38 years, two (25%, one female and one male) experienced sudden syncopes, four (50%, all female) were asymptomatic with QTc prolongation on ECG and one female (13%) was asymptomatic with normal findings on ECG (the *KCNQ1* variant was identified as an incidental finding in this individual). Apart from the proband who presented with sudden death, no other families had a history of sudden death in individuals < 45 years old.

**Table 1:** Comparison of clinical and electrophysiological phenotype of heterozygous *KCNQ1* variant carriers

	c.1124_1127delTTCA/p.(Ile375Argfs*43)	c.332A > G/p.(Tyr111Cys) [11]	c.1022C > T/p.(Ala341Val) [8]	c.1522C > T/p.(Arg518*) [12]	LQTS1/Non-p.(Ile375Argfs*43)	LQTS1/Non-(p.Ala341Val)
Female gender	22/41 (54%)	44/80 (55%)	132/244 (54%)	54/86 (63%)	15/19 (79%)	122/205 (60%)
Median FU, year (IQR)	45 (17–57)	23 (11–43)	30 (15–51)	NA	27 (11–43)	32 (14–46)
Mean age at last FU ± S.E.M. (y)	41 ± 22	28 ± 20	NA	29 ± 23	27 ± 18	NA
SCD	1/41 (2%)	1/80 (1%)	NA	1/86 (1%)	1/19 (5%)	NA
SCD < 40 y	1/41 (2%)	1/80 (1%)	74/244 (30%)***	NA	1/19 (5%)	14/205 (7%)
Symptomatic	3/41 (7%)	27/80 (34%)**	NA	15/86 (17%)	6/19 (32%)**	NA
Symptomatic < 40 y	2/41 (5%)	24/80 (30%)***	184/244 (75%)***	NA	6/19 (32%)**	49/205 (24%)**
QTc ≥ 500 ms	6/40 (15%)	19/78 (24%)	45/153 (29%)	20/81 (25%)	7/19 (37%)	26/190 (14%)
QTc ≤ 440 ms	13/40 (33%)	8/78 (10%)**	16/153 (10%)**	13/81 (16%)	3/19 (16%)	45/190 (24%)

Highest QTc measurements of resting ECGs were considered for the p.(Ile375Argfs*43) and LQTS1/Non p.(Ile375Argfs*43) populations. Statistical significance: **p < 0.01, ***p < 0.001, no *p*-value: not significant based on Fisher’s exact test

FU, follow up; IQR, interquartile range; NA, not available; S.E.M., standard error of mean

**Fig. 2:**
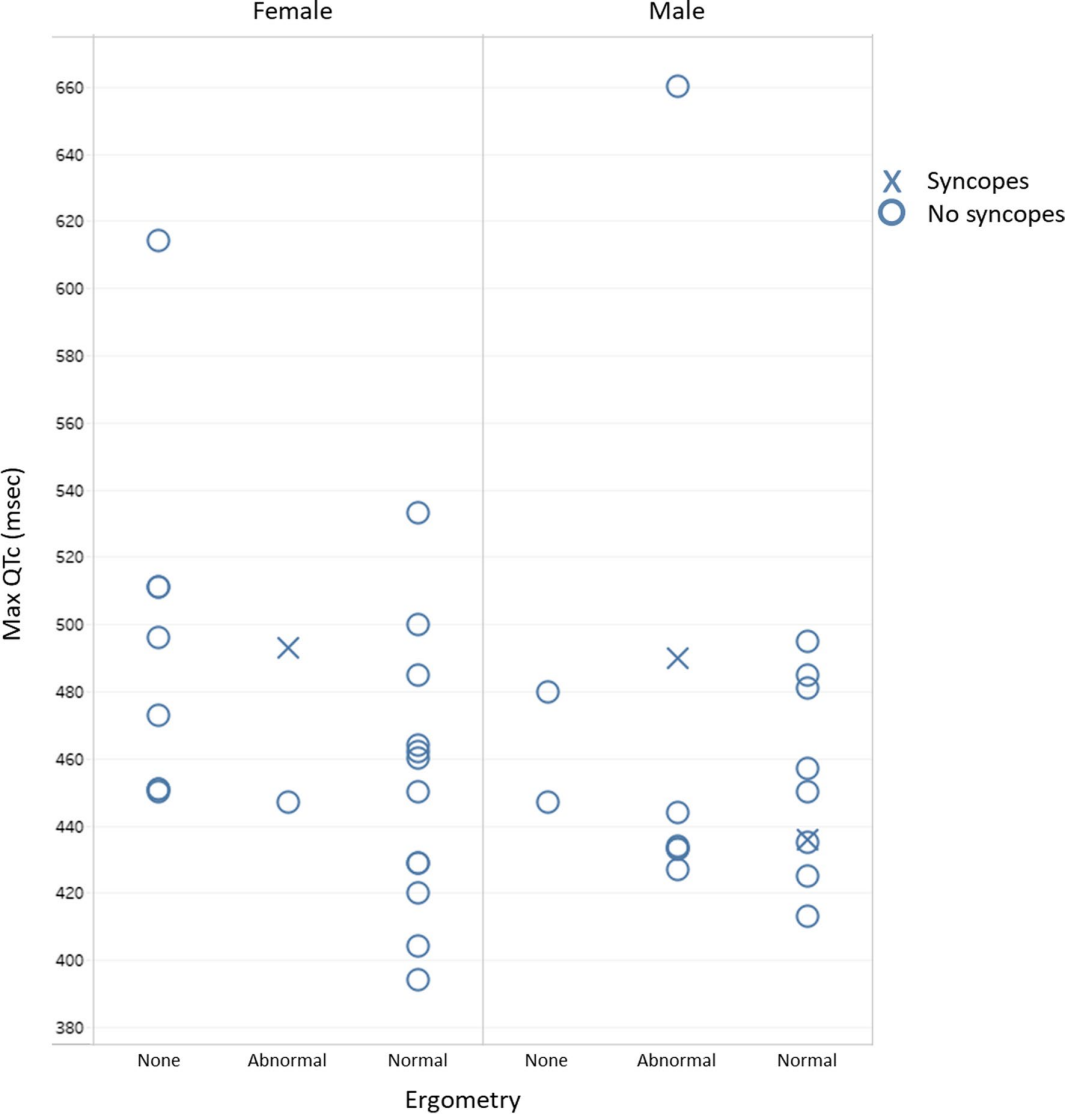
Clinical and electrophysiological phenotype of heterozygous c.1124_1127delTTCA p.(Ile375Argfs*43) variant carriers

One additional male heterozygous carrier experienced sudden syncope, resulting in a total of 3/41 (7%) heterozygous carriers with syncopes (Fig. 2). This individual was subsequently diagnosed with Brugada syndrome (BrS), based on a type 2 BrS ECG pattern, which converted to a type 1 pattern during the ajmaline provocation test. He had a maximum resting QTc of 436 ms and did not show QTc prolongation during exercise. No additional variants in other cardiac arrhythmia genes included in the PED MASTR Plus Assay (which includes *SCN5A*) were identified in this BrS patient. Five other variant harboring individuals from the same family underwent ajmaline provocation testing, none of them demonstrated a type 1 BrS pattern (even after ajmaline testing during exercise, to evaluate for rate-dependent effects). No spontaneous BrS ECGs were detected in any of the other families with the *KCNQ1* c.1124_1127delTTCA p.(Ile375Argfs*43) variant. Two of the heterozygous carriers who experienced syncopes despite beta-blocker treatment and in the absence of secondary causes were implanted with an internal cardioverter-defibrillator to prevent sudden arrhythmic death.

Maximum QTc values derived from resting 12-lead ECG recordings were available for 40 heterozygous carriers (55% female; not available for the SCD patient). The average maximal QTc interval was 466 ms (range 394–660 ms). For females (n = 22), the average QTc was 470 ms (range 394–614 ms for average age of 48 years (range 6–83 years)), for males (n = 18) 463 ms (range 413–660 ms for average age of 39 years (range 7–81 years)) (Fig. 2). Maximal QTc intervals measured on 24 h ECG holter recordings were available for 26 heterozygous carriers (53.8% female), with an average of 480 ms (range 430–540 ms). Of the 16/26 patients with a baseline QTc < 460 ms, 11 (69%) showed a QTc of > 460 ms on the 24 h ECG holter recording. One patient (3.8%) showed non-sustained ventricular tachycardia on 24 h ECG holter, all other patients did not demonstrate ventricular arrhythmia.

Out of 30 heterozygous variant carriers who had undergone an exercise stress test, nine (30%) showed an abnormal QT response to exercise (prolongation either during exercise or in the recovery phase, Fig. 2). When only the 16 patients who reached at least 80% of their predicted maximal heart rate (calculated as 220-age) were considered, an abnormal QTc response was observed in 38% of the variant carriers. Overall, 3/9 (33%) of variant harboring individuals with a resting QTc > 480 ms and 6/21 (29%) of those with a resting QTc ≤ 480 ms displayed an abnormal QT response to exercise. Six patients out of four different families (20%) showed significant depressions (> 1 mm, horizontal or downsloping) of the ST-interval upon exercise, while none of these patients showed signs of coronary disease upon additional testing. One patient (3.3%) displayed ventricular bigeminy during the exercise stress test, but no other forms of ventricular arrhythmia were detected.

To assess the severity of the clinical and electrophysiological phenotype of the heterozygous *KCNQ1* c.1124_1127delTTCA p.(Ile375Argfs*43) variant carriers, our data was compared to three publications on *KCNQ1* founder mutation populations, with a clinical spectrum ranging from rather benign for the Swedish p.(Arg518*) [12] and p.(Tyr111Cys) [11] mutations and remarkably severe for the South-African p.(Ala341Val) founder mutation [8] (Table 1). Additionally, a population of genetically heterogeneous non-p.(Ala341Val) *KCNQ1*-related LQTS patients described in the paper on the South-African founder mutation was included as comparison [8], as well as a population of genetically heterogeneous non-p.(Ile375Argfs*43) *KCNQ1*-related LQTS patients identified in our own center (Table 1). For the 19 non-p.(Ile375Argfs*43) *KCNQ1* pathogenic variant harboring individuals, the underlying variant was missense in 14 (74%), frameshift in 4 (21%) and splice site in one (5%) patient(s) (Additional file 2: Table S2).

We demonstrate a significantly lower incidence of symptoms in the c.1124_1127delTTCA p.(Ile375Argfs*43) population compared to the other groups, with the exception of the p.(Arg518*) variant, where only a non-significant trend was observed. Although both the p.(Ala341Val) and p.(Tyr111Cys) variants led to more symptoms, only the highly penetrant South-African p.(Ala341Val) founder mutation led to a significantly higher incidence of SCD (< 40 y), compared to our population. The p.(Ala341Val) and p.(Tyr111Cys) variants were also associated with a significantly lower proportion of individuals with normal (< 440 ms) QTc values (Table 1).

### Electrophysiological effect of the variant

**Fig. 3:**
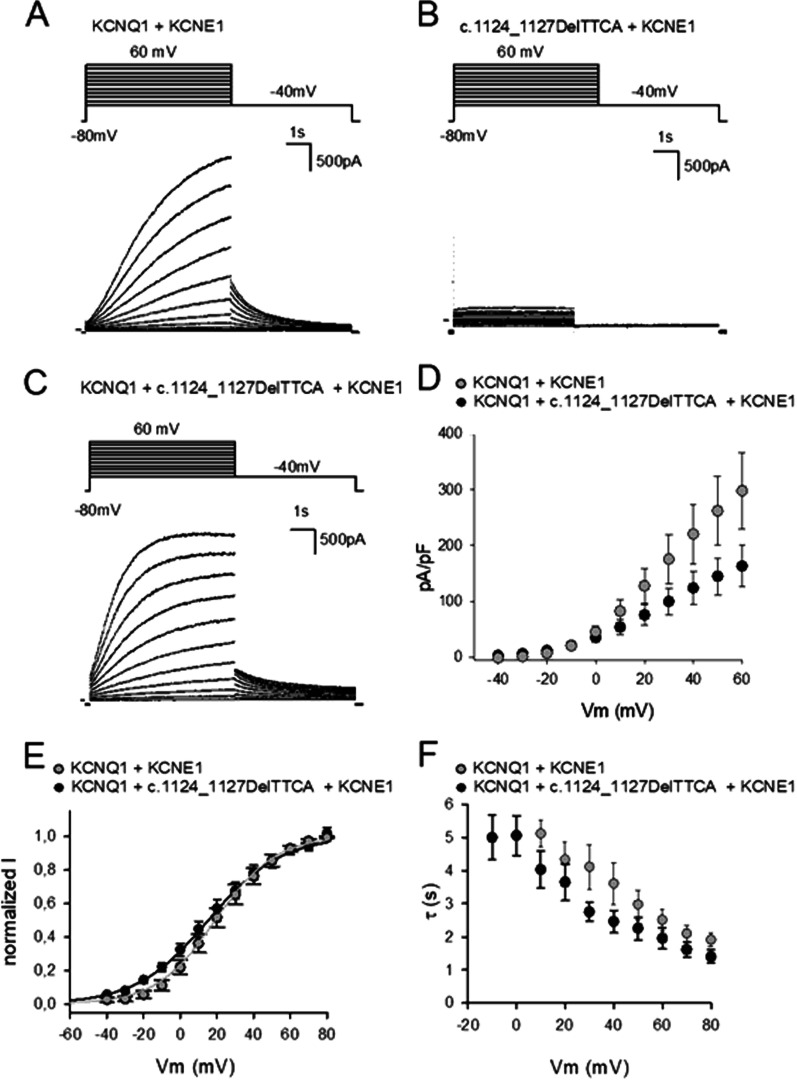
Results of electrophysiological experiments. **A**–**C** Representative current traces for CHO cells transfected with (**A**) *KCNQ1*/*KCNE1* (**B**) c.1124_1127delTTCA*/KCNE1* and (**C**) *KCNQ1*/c.1124_1127delTTCA/*KCNE1*. The voltage protocol is shown on top with below the corresponding current traces. **D** Current–voltage relationships for *KCNQ1*/*KCNE1* (n = 9, grey symbols) and *KCNQ1*/c.1124_1127delTTCA/*KCNE1* (n = 11, black symbols). Current amplitudes were determined at the end of a 6 s depolarizing step to + 60 mV. Current densities were calculated by dividing the current amplitudes by the cell capacitance. **E** Voltage-dependence of activation for *KCNQ1*/*KCNE1* (n = 7, grey symbols) and *KCNQ1*/c.1124_1127delTTCA/*KCNE1* (n = 6, black symbols). **F** Activation kinetics of *KCNQ1*/*KCNE1* (n = 7, grey symbols) and *KCNQ1*/c.1124_1127delTTCA/*KCNE1* (n = 6, black symbols). Shown values are the means ± standard error of mean with n the number of cells analyzed

Whole-cell patch clamp studies demonstrated no sizeable currents in CHO cells transfected with the c.1124_1127delTTCA construct together with *KCNE1* (Fig. 3B). However, when co-expressed with wildtype *KCNQ1*, the mutant channel complex generated *I*_*Ks*_-like currents (Fig. 3C), with reduced current densities compared to wildtype, consistent with a partial loss-of-function (Fig. 3D). On the other hand, the assessment of channel activation showed signs of accelerated activation kinetics for the mutant channel. The c.1124_1127delTTCA/*KCNQ1*/*KCNE1* complex displayed a slight, non-significant negative shift of the *V*_½_ (voltage at which 50% of the channels are activated), with a mean *V*_½_ of 15.3 mV (standard error of mean, S.E.M., of 3.8 mV, n = 6) for the mutant complex and a mean of 16.4 mV (S.E.M. of 4.7 mV, n = 7) for the wildtype (Fig. 3E). The slope of the activation curve fitted with the Boltzmann equation was slightly higher for the mutant complex (mean of 19.7 mV, S.E.M. of 1.8 mV, n = 6) compared to wildtype (mean of 16.5 mV, S.E.M. of 1.4 mV, n = 7) (p = 0.006 according to two sample t-test, Fig. 3E). A negative shift was also observed for the voltage-dependent activation time constants for the mutant channel complex (Fig. 3F).

### Gene expression

**Fig. 4:**
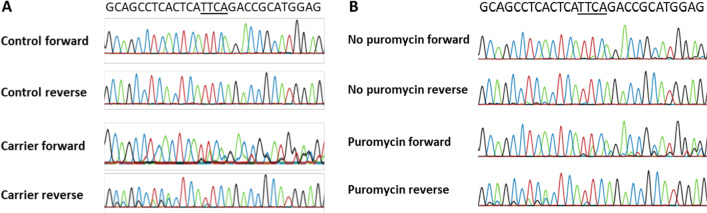
**A** Sanger sequencing of *KCNQ1* exon 8 on whole-blood derived cDNA from a heterozygous c.1124_1127delTTCA variant harboring individual (two bottom chromatograms) and a control individual (two top chromatograms). **B** Sanger sequencing of *KCNQ1* exon 8 on the same heterozygous c.1124_1127delTTCA variant carrier’s iPSC-CM derived cDNA, cultured without (two top chromatograms) and with (two bottom chromatograms) puromycin exposure. The c.1124_1127delTTCA site is underlined in the reference sequence. The whole-blood derived cDNA and iPSC-CM derived cDNA were obtained from the same individual

Sanger sequencing on cDNA extracted from whole blood of a heterozygous c.1124_1127delTTCA variant carrier showed expression of both the wildtype and c.1124_1127delTTCA allele (Fig. 4A), thereby suggesting that the frameshift allele escapes NMD. These findings were confirmed in a second individual from another family. Whole-blood derived cDNA from a healthy individual was used as a control.

The iPSC-CM expression assay was performed on cells obtained from one of the individuals who had previously displayed a lack of NMD in whole-blood derived RNA. Sanger sequencing on cDNA from iPSC-CMs derived from a heterozygous c.1124_1127delTTCA variant carrier showed nearly complete absence of the mutant allele. When the iPSC-CMs were exposed to puromycin (a NMD blocking agent), the mutant allele was expressed (Fig. 4B), thus proving that NMD occurred in iPSC-CMs.

## Discussion

We describe the identification of nine Belgian families with a pathogenic frameshift variant in *KCNQ1*, with initial presentation as Jervell-Lange-Nielsen syndrome in two siblings and LQTS in the remaining 8 families. Although we initially hypothesized that the high prevalence of this pathogenic variant in our region was caused by a founder effect, we were unable to demonstrate a shared haplotype for all nine families, even though two of the markers are located within the *KCNQ1* coding region. Surprisingly, the variant has previously only been described once in a cohort of 541 unrelated LQTS patients [22] and has an allele frequency of 1/251.100 according to GnomAD [20]. Given this low prevalence, it would also seem unlikely that the variant is located in a mutational “hot spot” region. Although improbable, we cannot exclude that potential founder effects have been masked by recombination events, or that a founder mutation occurred long ago and leads to a very small shared region (< 0.43 Mb), which is beyond the resolution of our haplotyping method. We did not find any molecular evidence that additional variants in any of the known arrhythmia genes explained the phenotypical variability that we observed in our cohort.

We found that 24 h ECG holter recordings added diagnostic value in unmasking QTc prolongation in patients with normal findings on conventional short term ECG recordings. This was suspected, as diurnal variation in QTc intervals, induced by e.g. adrenergic tone, can lead to false negative results with single ECG registrations [23]. Exercise stress testing also contributed to the diagnosis, with an abnormal QTc prolongation during or after exercise in approximately a third of our patient cohort. In addition, we observed significant ST-segment depressions on an exercise stress test, without evidence for cardiac ischemia on follow-up coronary artery evaluation, in 20% of heterozygous carriers. As false positive exercise stress tests appear to be relatively common (up to 18.8% of tested patients) [24], the added value of follow-up testing in patients referred for LQTS or other forms of inherited arrhythmia should be carefully considered to avoid unnecessary diagnostic workups. If further testing is considered, less invasive methods such as stress-echocardiography should be prioritized.

As allele-specific effects determine disease penetrance and expressivity for type 1 LQTS patients [13], we sought to further characterize the clinical phenotype of the c.1124_1127delTTCA p.(Ile375Argfs*43) variant. We compared our patient population with other cohorts of other (likely) pathogenic *KCNQ1* variants (either founder/recurrent mutations or a population of different *KCNQ1* variants). We showed that heterozygous c.1124_1127delTTCA p.(Ile375Argfs*43) variant harboring individuals display a significantly less symptomatic phenotype compared to the p.(Tyr111Cys) and the p.(Ala341Val) *KCNQ1* founder mutations, as well as other *KCNQ1* patients with different molecular defects, both published and followed up at our own center [8, 11].

We also observed a significantly higher proportion of patients with normal (< 440 ms) QTc segments compared to the p.(Tyr111Cys) and the p.(Ala341Val) *KCNQ1* founder mutations. The clinical and electrophysiological phenotype of our patients was most comparable to the Swedish p.(Arg518*) founder mutation, which is predicted to result in haploinsufficiency. It should be taken into account that the comparison between the different type 1 LQTS populations should be interpreted with care, as the data was extracted from separate studies with different inclusion criteria and methodology. Despite the generally mild phenotype, we also observed a single case of SCD in a 38 years old c.1124_1127delTTCA variant harboring individual. This individual had no additional genetic variants detected by the PED MASTR Plus Assay. He was asymptomatic prior to his death and had not previously undergone a cardiac evaluation.

As the Kv7.1 ion channel formed by the *KCNQ1* gene requires tetramerization to function, in-frame pathogenic variants which are expressed at the RNA and protein level can potentially result in a more severe phenotype by interfering with the co-assembly of wildtype proteins (dominant-negative effect). On the other hand, out-of-frame variants, which are expected to lead to RNA degradation and a lack of expression at the protein level, tend to present with a milder phenotype. The predominantly mild phenotype in carriers of the c.1124_1127delTTCA p.(Ile375Argfs*43) variant was most consistent with haploinsufficiency induced by NMD. However, surprisingly, initial expression studies based on whole-blood derived cDNA showed escape from NMD and retained expression of the mutant allele at the RNA level.

The finding of apparent NMD escape became particularly relevant when one symptomatic c.1124_1127delTTCA variant carrier in our cohort was diagnosed with BrS. *KCNQ1* variants have only been reported in association with BrS on an incidental basis [25]. Pathogenic variants in other potassium channels, most notably *KCNH2*, have been described as contributing to the BrS phenotype through gain-of-function mechanisms [26]. As the c.1124_1127delTTCA allele appeared to be expressed, we decided to perform electrophysiological characterization to investigate its potential contribution to the BrS phenotype in our patient.

The LQTS/BrS overlap hypothesis indeed initially seemed supported by evidence for both loss-of-function (decreased current density) and gain-of-function (acceleration of channel kinetics) effects in CHO cells [27]. However, despite additional sodium blocker challenge testing in this family, BrS was not identified in other variant carriers. Additionally, at a later timepoint, the children of the BrS patient, who were not carrying the *KCNQ1* variant, were diagnosed with BrS based on a positive sodium blocker challenge test. Subsequent expression studies in iPSC-CMs further disproved the overlap theory by demonstrating near complete degradation of the mutant allele via NMD. These findings refute a dominant role for the *KCNQ1* variant in the pathogenesis of BrS in our patient. However, we did not assess *KCNQ1* expression in either iPSC-CMs or cardiac tissue of this BrS presenting individual. As little is known about inter-individual variability in NMD, we cannot exclude that the variant could still play a modifying role on the BrS phenotype.

Studies on large patient groups carrying the same pathogenic variant are valuable for both the characterization of potential allele specific effects and other genetic factors (e.g. modifiers, additional genetic variants identified in our cohort are summarized in Additional file 1: Table S1) [28], which play a role in disease expressivity in LQTS. These populations have also provided an interesting look into the correlation between functional variant characterization and the clinical phenotype. Discrepancies have been described for the relatively mild dominant-negative effect of the p.(Ala341Val) variant in vitro, compared to the severe clinical phenotype [8, 10]. The opposite effect was observed for the p.(Tyr111Cys) variant, with a strong dominant-negative effect in vitro and relatively mild clinical phenotype [11, 29]. In the cardiomyocyte, the variant’s effect can be influenced by other factors, such as accessory proteins, other ion channels or RNA and protein processing pathways. The inability of heterologous systems to fully recapitulate this complex cellular environment is a plausible explanation for these discrepant findings.

For our c.1124_1127delTTCA p.(Ile375Argfs*43) variant, the electrophysiological characterization indeed confirmed decreased current density, as expected for haploinsufficiency. However, despite initial expectations, the acceleration of channel kinetics induced by the variant in vitro may not be clinically relevant. It is possible that the apparent kinetic effect is induced artificially by partially retained expression of the mutant allele due to the high dose of mutant mRNA generated by the heterologous overexpression. The finding of differing NMD activity in whole blood and iPSC-CMs also sheds doubt on the representativeness of NMD or potentially other RNA-based mechanisms such as splicing, in unaffected tissues or heterologous expression models.

Unfortunately, complete clinical characterization is unfeasible for most sporadic *KCNQ1* variants due to a low number of variant carriers, although this could be improved by increased data sharing and collaborations. In situations with lack of clinical data, functional studies remain the main alternative for variant classification. It will be particularly interesting to investigate whether electrophysiological examinations of iPSC-CMs offer a more clinically accurate representation of the patients’ phenotypes, compared to heterologous expression. Studies on iPSC-CMs derived from carriers of *KCNQ1* founder mutations will likely contribute to this goal [30]. Although iPSC-CMs still suffer from problems with maturity and variability [31], which might hamper their role in electrophysiological phenotyping, they constitute a very promising model.

The inability to confirm the expression of the mutant allele in native cardiomyocytes remains a limitation of this study. Nonetheless, we expect that iPSC-CMs represent the RNA processing mechanisms in cardiomyocytes more reliably than other tissues (such as blood leukocytes). As we only performed the iPSC-CM expression studies in cells derived from a single heterozygous variant carrier, we were unable to assess if NMD would function differently between individual variant carriers. As the generation of patient derived iPSC-CMs remains both expensive and labor intensive, for the moment it is not feasible as a tool for functional evaluation of all variants which might undergo NMD or lead to splicing defects. Hopefully, improvements and/or cost reduction (e.g. due to automatization) in the creation of iPSCs and/or in our ability to generate new variants in control iPSC lines by gene editing methods such as CIRSPR-Cas9, will enable a more performant characterization of novel variants in the future.

## Conclusions

The c.1124_1127delTTCA p.(Ile375Argfs*43) variant in the *KCNQ1* gene shows a high prevalence in our region, despite remaining unconfirmed as a founder mutation. Clinically, this variant leads to Jervell-Lange-Nielsen syndrome in the homozygous state and a predominantly mild LQTS phenotype in heterozygous patients. We have demonstrated the mild clinical effect of the variant by comparing the clinical phenotype of our patients with carriers of other (likely) pathogenic *KCNQ1* (founder) mutations. We found that despite initial evidence for NMD escape in blood and a mixed loss- and gain-of-function effect in CHO functional studies, additional testing in iPSC-CMs showed almost complete lack of expression of the c.1124_1127delTTCA allele, more consistent with haploinsufficiency. Based on our findings, we recommend caution when linking functional data to the clinical phenotype, especially for gene expression studies on clinically unaffected tissues such as blood. Although we did not find evidence for pathogenic “second hits” in any of the known arrhythmia genes, we cannot rule out such genetic modifiers as an explanation for the phenotypical variability.

## Supplementary Information

**Additional file 1: Table S1**. Demographics, clinical and genetic data of the c.1124_1127delTTCA p.(Ile375Argfs*43) *KCNQ1* variant carriers

**Additional file 2: Table S2**. *KCNQ1* variants included in the non-p.(Ile375Argfs*43) *KCNQ1* group

## Data Availability

The clinical data used for the generation of this paper is included in the supplement. All other datasets used and/or analysed during the current study are available from the corresponding author on reasonable request.

## Abbreviations

LQTS: Long QT syndrome
BrS: Brugada syndrome
iPSC-CMs: Induced pluripotent stem cell cardiomyocytes
QTc: Corrected QT interval
ECG: Electrocardiogram
*I*_*Ks*_: Delayed rectifier potassium current
NMD: Nonsense mediated mRNA decay
STRs: Short tandem repeats
FAM: 6-Fluorescein amidite
SNP: Single nucleotide polymorphism
SCD: Sudden cardiac death
CHO: Chinese hamster ovary
MBP: Mega base pairs
FU: Follow up
IQR: Interquartile range
NA: Not available
S.E.M.: Standard error of mean
