# Supplementary material for: Clinical and functional characterisation of a recurrent KCNQ1 variant in the Belgian population
Source: Orphanet J Rare Dis. 2023 Jan 31;18:23. doi: 10.1186/s13023-023-02618-4 (PMC9887867; doi:10.1186/s13023-023-02618-4)
Supplement: Supplementary file 2 — Additional file 2: Table S2. KCNQ1 variants included in the non-p.(Ile375Argfs*43) KCNQ1 group [file 13023_2023_2618_MOESM2_ESM.docx]

**Additional file 2: Table S2**. *KCNQ1* variants included in the non-p.(Ile375Argfs*43) *KCNQ1* group

| **Coding DNA reference** | **Protein reference** | **Number of patients** | **N female (%)** | **Mean age at diagnosis (range)** |
| --- | --- | --- | --- | --- |
| c.364dupT | p.(Cys122Leufs162*) | 2 | 2 (100%) | 23 (19-27) |
| c.535G>A | p.(Gly179Ser) | 1 | 0 (0%) | 27 |
| c.691C>T | p.(Arg231Cys) | 4 | 4 (100%) | 27 (4-57) |
| c.1022C>T | p.(Ala341Val) | 4 | 4 (100%) | 18 (5-44) |
| c.1031C>T | p.(Ala344Val) | 1 | 1 (100%) | 33 |
| c.1032G>A | (p.=) (splice site mutation) | 1 | 1 (100%) | 58 |
| c.1096C>T | p.(Arg366Trp) | 3 | 1 (33%) | 12 (3-31) |
| c.1097G>A | p.(Arg366Gln) | 1 | 1 (100%) | 45 |
| c.1262_1265delAAAA | p.(Lys421Serfs*10) | 1 | 0 (0%) | 42 |
| c.1349delA | p.(Glu450Glyfs*?) | 1 | 1 (100%) | 47 |
